# Effect of cytokines on Siglec-1 and HIV-1 entry in monocyte–derived macrophages: the importance of HIV-1 envelope V1V2 region

**DOI:** 10.1189/jlb.2A0815-361R

**Published:** 2015-12-14

**Authors:** Ousman Jobe, Hung V. Trinh, Jiae Kim, Wadad Alsalmi, Sodsai Tovanabutra, Philip K. Ehrenberg, Kristina K. Peachman, Guofen Gao, Rasmi Thomas, Jerome H. Kim, Nelson L. Michael, Carl R. Alving, Venigalla B. Rao, Mangala Rao

**Affiliations:** * Henry M. Jackson Foundation for the Advancement of Military Medicine, Silver Spring, Maryland, USA;; † Laboratory of Adjuvant and Antigen Research, U.S. Military HIV Research Program, Walter Reed Army Institute of Research, Silver Spring, Maryland, USA;; § Laboratory of Molecular Virology and Pathogenesis, Viral Sequencing Core, U.S. Military HIV Research Program, Walter Reed Army Institute of Research, Silver Spring, Maryland, USA; and; ¶ Host Genetics Section, U.S. Military HIV Research Program, Walter Reed Army Institute of Research, Silver Spring, Maryland, USA; and; ‡ Department of Biology, The Catholic University of America, Washington, DC, USA

**Keywords:** GM-CSF, M-CSF, qRT-PCR, viral entry, flow cytometry, surface plasmon resonance

## Abstract

Monocytes and monocyte–derived macrophages express relatively low levels of CD4. Despite this, macrophages can be effectively infected with human immunodeficiency virus type 1. Macrophages have a critical role in human immunodeficiency virus type 1 transmission; however, the mechanism or mechanisms of virus infection are poorly understood. We report that growth factors, such as granulocyte macrophage colony-stimulating factor and macrophage colony-stimulating factor affect the phenotypic profile and permissiveness of macrophages to human immunodeficiency virus type 1. Human immunodeficiency virus type 1 infection of monocyte–derived macrophages derived from granulocyte macrophage and macrophage colony-stimulating factors was predominantly facilitated by the sialic acid-binding immunoglobulin-like lectin-1. The number of sialic acid-binding immunoglobulin-like lectin receptors on macrophage colony-stimulating factor–derived monocyte–derived macrophages was significantly greater than on granulocyte macrophage colony-stimulating factor–derived monocyte–derived macrophages, and correspondingly, human immunodeficiency virus type 1 infection was greater in the macrophage colony-stimulating factor–derived monocyte–derived macrophages. Single-genome analysis and quantitative reverse transcriptase-polymerase chain reaction revealed that the differences in infectivity was not due to differences in viral fitness or in viral variants with differential infectivity but was due to reduced viral entry into the granulocyte macrophage colony-stimulating factor–derived monocyte–derived macrophages. Anti-sialic acid-binding immunoglobulin-like lectin, trimeric glycoprotein 145, and scaffolded V1V2 proteins were bound to sialic acid-binding immunoglobulin-like lectin and significantly reduced human immunodeficiency virus type 1 entry and infection. Furthermore, sialic acid residues present in the V1V2 region of the envelope protein mediated human immunodeficiency virus type 1 interaction with sialic acid-binding immunoglobulin-like lectin and entry into macrophage colony-stimulating factor–derived monocyte–derived macrophages. Removal of sialic acid residues or glycans from scaffolded V1V2 protein decreased human immunodeficiency virus type 1 infectivity. These results highlight the importance of sialic acids on the V1V2 region in binding to sialic acid-binding immunoglobulin-like lectin and suggest that the unusually long surface-exposed sialic acid-binding immunoglobulin-like lectin might aid in the capture and entry of human immunodeficiency virus type 1 into monocyte–derived macrophages.

## Abstract (precis)

M-CSF increased Siglec-1 expression on macrophages, rendering them more permissive to HIV-1 infection due to interaction with V1V2 region of gp120 and associated sialic acids.

## Introduction

Macrophages are targets of HIV-1 and represent a potentially long-lived viral reservoir. In humans, macrophages arise from circulating or resident monocytes, and their differentiation and effector functions are largely dependent on the surrounding microenvironment [1, 2]. Generally, macrophages are classified into 2 types, classically activated (M1) and alternatively activated (M2) macrophages. Following in vitro stimulation with various stimuli, M2 macrophages are further divided into 3 subtypes (M2a, M2b, and M2c) [3]. Macrophages exhibit phenotypic heterogeneity that is dependent upon the cytokines present within their environment [4, 5]. GM-CSF and M-CSF are macrophage growth factors that have distinct effects on macrophage differentiation, resulting in either the M1 or the M2 phenotype [6]. M1 macrophages produce IL-1β, IL-12, IL-23, and TNF-α, as well as reactive oxygen and nitrogen intermediates; support Th1 responses; and mediate resistance to tumors and intracellular pathogens. M2 macrophages secrete IL-10, express scavenger and mannose receptors, contribute to Th2 responses, enhance phagocytosis, eliminate parasites, and promote tissue repair [7, 8].

CD4 molecules and chemokine receptors are the major receptor/coreceptors used by HIV-1 for infection of target cells. However, unlike CD4^+^ T cells, macrophages and MDMs express relatively low levels of surface CD4, thus, confounding the role of this molecule in HIV-1 entry in macrophages. Other unique cellular molecules, such as α4β7 [9, 10], tetraspanins [11], and heparan sulfate proteoglycans [12], have a role in HIV-1 infection. Recently, Siglec-1 was shown to facilitate HIV-1 infection of DCs [13, 14] and macrophages [15] by binding to the sialoglycans on the gp120 envelope. To date, 15 different Siglec receptors have been characterized in humans, and these proteins specifically recognize the terminal SAs associated with both *N*- and *O*-linked glycosylation promoting cell-to-cell adhesion [16]. The viral envelope of HIV is heavily glycosylated, and SAs on the viral envelope interact with Siglec receptors, in particular Siglec-1 (CD169), on macrophages and DCs, facilitating HIV-1 infection. Siglec-1 on DCs capture HIV-1 by interacting with sialyllactose-containing gangliosides exposed on viral membranes and subsequently mediate *trans*-infection of CD4^+^ T cells [13, 17]. In the case of macrophages, it is not known which region of the HIV-1 envelope protein interacts with Siglec-1 or whether the conformation of the viral envelope protein is also important in that interaction.

In the present study, we evaluated the effects of GM-CSF and M-CSF on the expression of Siglec receptors on primary human MDMs, the relationship between Siglec receptors and the permissiveness of HIV-1 infection, and the interaction of Siglec-1 with HIV-1 trimeric envelope protein. We further investigated whether the V1V2 region of HIV-1 gp120 protein interacted with Siglec-1 and how that interaction influenced HIV-1 infectivity.

## MATERIALS AND METHODS

### Ethics statement

RV229B (WRAIR protocol 1386) “Apheresis of blood components from healthy volunteers for in vitro research” and all related documents were approved by the following independent institutional review boards: the Division of Human Subject Protection, the WRAIR, and the Ethical Review Committee for Research in Human Subjects. All volunteers were provided written, informed consent after discussion and counseling by the clinical study team before enrollment and before any blood was drawn.

### Antibodies

The human mAbs anti-CD1a APC (clone HI149), CD11b PE (clone ICRF44), CD11b FITC (clone ICRF44), CD4 PE (clone RPA-T4), CD4 purified (clone RPA-T4), Siglec-3 (CD33)-APC (clone WM53), CD206 APC (clone 19.2), CD14 APC (clone M5E2), CD14 PerCP (clone MoP9), CD33 purified (clone WM53), CD4 PE (clone SK3), CD4 purified (SK3), CD195 PE (clone 2D7/CCR5), CD195 purified (clone 2D7/CCR5), HLA-A,B,C (clone G46-2.6), and 7-amino actinomycin D were obtained from BD Pharmingen (San Jose, CA USA). Purified Siglec-1, Siglec-5, and Siglec-9 mAbs, and the corresponding Siglec mAbs with the fluorochromes anti-Siglec-1 (CD169)-APC (clone 7-239), Siglec–9 PE (clone K8), and Siglec-5 (CD170)-PE (clone 1A5), were obtained from BioLegend (San Diego, CA, USA). Anti-HLA-DR (clone L203) was obtained from R&D Systems (Minneapolis, MN, USA). Anti-p24-FITC and anti-p24-RD1 were purchased from Beckman Coulter (Indianapolis, IN, USA).

### Media and reagents

Media components and reagents were obtained as follows: RPMI 1640; l-glutamine, penicillin/streptomycin, and EDTA (Quality Biologicals Inc., Gaithersburg, MD, USA); Accutase (eBioscience, San Diego, CA, USA); recombinant human M-CSF and GM-CSF (PeproTech, Inc, Rocky Hill, NJ, USA); and FBS (Gemini Bio Products, West Sacramento, CA, USA). BSA, Nonidet P-40, Polybrene, Triton X-100, SDS, dual 5′ FAM-labeled and 3′ TAMRA-labeled probes and amplifying primers, and dual 5′ FAM were obtained from Sigma-Aldrich (St. Louis, MO, USA). Fixation and permeabilization buffer reagents A and B were from Life Technologies (Frederick, MD). Proteinase K, 2X GeneAMP Fast PCR Master Mix, 2X TaqMan Universal PCR Master Mix, and dual 5′ VIC-labeled and 3′ TAMRA-labeled probes were purchased from Applied Biosystems (Foster City, CA, USA). Tris-HCl and Tween-20 were purchased from Invitrogen (Carlsbad, CA, USA).

### Peptides

Cyclic peptides were synthesized by JPT Peptide Technologies (Berlin, Germany). Peptides were cyclized by disulfide bond formation, and the purity was determined to be >90% by HPLC and mass spectrometry. The amino acid sequence of cyclic V2 peptides was based on vCP1521 envelope gp of HIV-1 CRF01 AE (92TH023 strain), GenBank accession number EF553537.1, and has been previously described [18].

### Construction, expression, and purification of recombinant HIV-1 envelope proteins

JR-FL and SF162 gp120 and trimeric gp145 were produced in 293F cells. V1V2 proteins and CHO-S cells were also produced in these cells as either Soc or as gp16-scaffolded proteins. Soc is a nonessential Soc of bacteriophage T4, and gp16 is an 18-kDa oligomeric complex required for packing T4 DNA [19].

Soc and T4 gp16 were codon-optimized using GeneArt gene synthesis by Life Technologies (Carlsbad, CA, USA). In the N terminus, a GLuc secretion signal from Gaussia luciferase protein was included to allow for sufficient protein secretion, and a hexahistidine tag was included in the C terminus to facilitate protein purification. The V1V2 loop of SF162, JR-FL, and the transmitted/founder virus Zm249 were constructed using gene assembly PCR [20]. Restriction sites *BamHI* and *EcoRI* were included in the N and C terminus of the V1V2 loop, respectively. The fragments were then cloned into a linearized vector containing either a GLuc-RB69soc-*BamHI*-*EcoRI*-hexahistadine tag or a GLuc-T4gp16-*BamHI*-*EcoRI*-hexahistadine tag. JR-FL and SF162 gp145 DNA were provided by Dr. Peter Kwong (National Institutes of Health, Bethesda, MD, USA). The codon-optimized constructs contain a CD5 secretion signal, complete gp120 and gp41 ectodomains, a mutation in the proteolytic cleavage site, and a trimerization domain from bacteriophage T4 fibritin [21] in the C terminus. JR-FL and SF162 gp120 were constructed from gp145 clones using PCR. All constructs were ligated with the linearized and dephosphorylated pcDNA3.1 vector (Invitrogen).

Transient transfection was performed using linear polyethylenimine (PEI25k, Polysciences, Inc., Warrington, PA, USA). Briefly, suspension cells 293F and CHO-S were grown per manufacture’s suggestions to maintain cells in their exponential growth phase. Two hours before transfection, the cell suspension was centrifuged (100 *g* for 5 min) and resuspended in fresh medium at 3 × 10^6^ cell/ml. Cells were then transfected with 1 μg DNA/10^6^ cells at a polyethylenimine to DNA ratio of 3:1. The following day, media was added to the cells to bring the cell concentration to 1 × 10^6^ cell/ml. Transfection supernatants were harvested on day 3 and clarified using a 0.2-μm filter.

The clarified supernatant was loaded onto a HisTrap column (GE Healthcare, Little Chalfont, United Kingdom) and washed with buffer containing 20 mM imidazole. The protein was eluted with a 20–300-mM linear imidazole gradient. Soc–V1V2 loop peak fractions were concentrated using a 10-kDa molecular weight cutoff Amicon Ultra-4 centrifugal filter unit (Millipore, Billerica, MA, USA), and buffer was exchanged in PBS (pH 7.4) and stored at −80°C. The gp145 and gp120 peak fractions were concentrated and applied to a Hi-Load 16/60 Superdex-200 (preparation grade) gel filtration column (GE Healthcare) equilibrated in 20 mM Tris-HCl (pH 8.0) and 100 mM NaCl. The peaks corresponding to a trimeric gp145 and monomeric gp120 were identified, pooled, concentrated, and stored at −80°C.

### Desialylation and deglycosylation of Soc–V1V2

Purified Soc–V1V2 Zm249 scaffold protein (10 µg) was desialylated by treatment with 60 units of α2-3,6,8,9 neuraminidase A in 50 mM sodium acetate buffer (pH 5.5) containing 5 mM CaCl_2_ or deglycosylated with 1500 units of PNGase F (New England BioLabs Inc., Ipswich, MA, USA) in 50 mM sodium phosphate buffer (pH 7.5). The samples were incubated for 5 h at 37°C. The control samples lacked the neuraminidase A or PNGase F treatment.

To remove the enzymes, the treated and control samples were incubated with Ni-NTA agarose beads (Qiagen, Valencia, CA, USA) and placed on a rotating platform for 3 h at 4°C. The bead mixture was then spun down in a Pierce spin column (Thermo Fisher Scientific, Rockford, IL, USA), and the flow-through fraction was collected. The beads captured the His-tagged Soc–V1V2 protein, whereas the free neuraminidase A and PNGase F enzymes eluted in the flow-through fraction. To remove any residual enzyme, the beads were washed twice with an excess of 50 mM Tris-HCl buffer (pH 8.0), containing 300 mM NaCl. Bound Soc–V1V2 proteins were then eluted with 300 mM imidazole, concentrated, and buffer exchanged (25 mM Tris-HCl buffer [pH 8.0] containing100 mM NaCl) using 10-kDa MW cutoff Amicon Ultra-4 centrifugal filter units (Millipore).

Untreated neuraminidase A and PNGase F–treated Soc–V1V2 Zm249 scaffold proteins, before and after removal of the respective enzymes from the reaction mixture, were run on a 4–20% gradient Tris-glycine gel (Life Technologies) under reducing conditions. The gels were stained with Coomassie brilliant blue. Treatment with neuraminidase A or with PNGase F resulted in desialyation and deglycosylation as visualized by a shift in the mobility of the protein on SDS-PAGE with the proteins showing sharper bands after enzymatic treatment compared with the diffused bands before treatment. Neuraminidase A and PNGase F were removed from the reaction mixtures by using Ni-NTA agarose beads, which specifically bound to His-tagged Soc–V1V2, allowing the enzymes to pass in the flow-through fractions.

### Virus purification

Virus stocks of US-1 and BaL (HIV-1 primary isolates subtype B), MO66 (primary isolate subtype CRF01_AE), and TZBD9/11 (primary isolates subtype C) grown in PBMCs were provided by Dr. Victoria Polonis (U.S. Military HIV Research Program, Silver Spring, MD, USA). The viruses were purified as previously described [22]. Infectivity and p24 concentrations were determined before and after purification to ensure that infectivity was not lost during the purification procedure.

### Enrichment and in vitro culture of monocytes

PBMCs were isolated by Ficoll density gradient centrifugation from healthy HIV-1–seronegative donors under an internal review board–approved protocol (RV229/WRAIR 1386). Monocytes were enriched from the PBMCs by plastic adherence. Briefly, PBMCs were resuspended in serum-free monocyte attachment media (RPMI 1640 supplemented with 1% l-glutamine and 1% penicillin/streptomycin), transferred to a tissue culture flask (CoStar Group, Washington DC, USA), and incubated at 37°C/5% CO_2_ for 1 h. The nonadherent cells were aspirated, and the monolayer was washed with monocyte media (RPMI 1640 supplemented with 10% heat-inactivated FBS, 1% l-glutamine, and 1% penicillin/streptomycin) to remove the remaining nonadherent cells. Accutase was added and the flask was incubated at 37°C/5% CO_2_ for 30 min. Most of the cells are detached at that time, and gentle pipetting detached the remaining cells. Cells were washed, counted, and analyzed by flow cytometry or plated in 24-well plates. Routine flow cytometric analysis with anti-CD14, anti-CD3, anti-CD11b, and 7-amino actinomycin D for monocyte characterization showed a purity of >90% and viabilities of >98%, with no T cell contamination. Similar viabilities were also observed with trypan blue exclusion. The enriched monocytes were subsequently differentiated into MDMs in monocyte medium supplemented with M-CSF or GM-CSF medium. Both growth factors were used at a final concentration of 50 ng/ml. For evaluation of surface markers, MDMs were harvested on days 1, 3, and 5 postculture; stained; and analyzed by flow cytometry. For infection with HIV-1, the MDMs were used on day 5 postculture.

### HIV-1 infection of MDMs

MDMs differentiated in M-CSF medium or in GM-CSF medium were plated at 0.5 × 10^6^ cells per well in 24-well plates (CoStar), as described above, and infected with HIV-1 as previously described [22]. Briefly, the cells were incubated with 300 μl of infection media (monocyte medium containing 2 μg/ml Polybrene) at 37°C/5% CO_2_ for 30 min. Following aspiration, 300 μl of fresh infection medium containing purified HIV-1 (5 ng p24/300 μl) or envelope-deficient HIV-1 (pSG3^Δenv^) (5 ng p24/300 μl) was added to each monolayer, and the plate was centrifuged at 2500 rpm for 90 min at 37°C. pSG3Δenv contained a 4-nucleotide insertion mutation (CTAG) in the envelope, leading to a translation stop codon after 142 aa residue. The pSG3Δenv clone is routinely used for generating envelope pseudo-typed infectious virions. Unadsorbed virus was removed, and the cells were incubated at 37°C/5% CO_2_ in 1 ml of M-CSF or GM-CSF medium containing 2 μg/ml of Polybrene. Culture supernatants and cells were harvested at predetermined time points (15 and 30 min and 1, 3, 24, and 96 h postinfection for entry assays; 2, 4, and 10 d postinfection for infectivity assays). Supernatants were stored at −20°C. Cells were harvested and stained, and the percentage of HIV-1–infected cells (p24^+^ MDMs) was determined by flow cytometry.

### Detection of cell-surface molecules

MDMs were harvested and washed in cold FACS buffer (PBS containing 0.5% BSA). The cells were incubated for 20 min at 4°C with a cocktail containing 5–10 μg of the specific mAb or its corresponding isotype controls. Cells were washed in cold FACS buffer and fixed in PBS containing 2% paraformaldehyde. Four-color flow cytometry was performed on a FACSCalibur cell analyzer (BD Biosciences). Data analyses were performed on the gated CD14^+^ cells using FlowJo 8.8.6 software (Tree Star Inc.).

### Detection of intracellular HIV-1 p24 antigen

Briefly, HIV-1-infected and uninfected MDM were resuspended in cold FACS buffer (PBS-containing 0.5% BSA) and incubated with 5-10 μg anti-CD14 mAb for 20 min at 4°C. Cells were washed in cold FACS buffer and fixed in 100 μl Reagent A, in the dark, for 15 min at room temperature. Following washing in FACS buffer, the cells were permeabilized with 100 μl Reagent B and stained with anti p24-FITC or anti p24-PE for 15 min at room temperature. Cells were washed in cold FACS buffer and resuspended in PBS. Flow cytometry was performed on a FACSCalibur system. Data analyses were performed on the gated CD14^+^ cells using FlowJo 8.8.6 software (Tree Star, Ashland, OR, USA).

### Quantification of cell-surface receptors

The number of Siglec-1, CCR5, or CD4 receptors/cell was determined using Quantum Simply Cellular Beads (Bangs Laboratories, Inc., Fishers, IN, USA) according to the manufacturer’s instructions. Briefly, MDMs (0.5 × 10^5^ cells/tube) were preincubated with 10% normal goat sera, followed by the addition of a mAb cocktail of anti-human Siglec-1 (CD169)–APC, anti-human CCR5 (CD195)–FITC, or anti-human CD4-PE for 30 min at 4°C. Cells were then fixed with formaldehyde. Beads from the kit were stained with anti-human Siglec-1 (CD169)–APC, anti-human CCR5 (CD195)–FITC, or anti-human CD4-PE. Individual standard curves were established using the stained beads. Samples were acquired on a FACSCalibur system and analyzed with FlowJo 8.8.6 software. Data were placed into QuickCal version 2.3 software (Bangs Laboratories), and the number of Siglec-1, CCR5, or CD4 receptors/cell was extrapolated from the standard curves generated with the stained beads.

### Blocking experiments

M-CSF–derived or GM-CSF–derived MDMs were plated at 0.5 × 10^6^ cells/well in 24-well plates. The cells were preincubated with 300 μl of purified mAbs (10 μg/ml), *N*-acetylneuraminic acid (10 mM SA; Carbosynth, Compton, West Berkshire, United Kingdom), lactose (50 mM), sialyllactose (GM3, 10 mM), recombinant-scaffolded HIV-1–V1V2 proteins (20 μg/ml), PNGase-treated recombinant HIV-1–V1V2 proteins (20 μg/ml), neuraminidase A–treated recombinant HIV-1–V1V2 proteins (20 μg/ml), or varying concentrations of JR-FL FD gp145 trimer for 30 min at room temperature. Following aspiration and washing, 300 μl of fresh infection medium containing purified HIV-1 was added to each monolayer, and the plate was centrifuged at 2500 rpm for 15 or 30 min at 37°C (for HIV-1 entry experiments) or for 90 min at 37°C (for HIV-1 replication experiments). For HIV-1 entry experiments, unadsorbed virus was removed, and the cells were washed 3 times with PBS to remove residual virus. The cells were subsequently lysed, and HIV-1 entry was determined by qRT-PCR or by qPCR. For HIV-1 replication, unabsorbed virus was removed, and the cells were incubated at 37°C/5% CO_2_ in 1 ml of M-CSF or GM-CSF medium containing Polybrene. Cells were harvested and stained, and the percentage of HIV-1–infected cells was determined by flow cytometry.

### SPR measurements

Surface plasmon measurements were conducted with a Biacore T200 (GE Healthcare). Siglec-1 was immobilized onto a CM5 sensor chip using a standard amine-coupling method. *Escherichia coli* expressed recombinant T4gp15 protein or Fc–mucosal addressin cell adhesion molecule-1 protein was immobilized on the reference flow cell. Briefly, the chip surface was activated by a 1:1 mixture of 0.4 M 1-ethyl-3-(3-dimethylaminopropyl) carbodiimide hydrochloride and 0.1 M *N*-hydroxysuccinimide for 7–10 min with a flow rate of 10 µl/min. Siglec-1 (2500 RUs) and T4gp15 (3100 RUs) were coupled. The immobilized surface was deactivated with 1.0 M ethanolamine–HCl (pH 8.5) for 10 min and stabilized with multiple injections of 0.05% SDS and 50 mM HCl. In separate experiments, JR-FL monomeric gp120 (subtype B, 900 RUs), JR-FL FD trimeric gp145 (1400 RUs), SF162 FD trimeric gp145 (subtype B, 900 RUs), Soc–V1V2JR–FL (1150 and 8000 RUs), and Soc–V1V2SF162 (1150 RUs) were immobilized on different CM5 chips using the same standard amine-coupling procedure described above.

Varying concentrations (0.98–1000 nM) of gp120, gp145, and Soc–V1V2 proteins, or alternatively, Siglec-1 in 10 mM HEPES and 150 mM NaCl (pH 7.4) were injected over immobilized Siglec-1 or gp120/gp145/Soc–V1V2 proteins, respectively, at a flow rate of 30 µl/min, for 2 min, followed by a dissociation time of 8 min. The chip surface was regenerated with 0.05–0.075% SDS for 30–45 s. Each kinetic assay was performed in triplicate. The data were analyzed using the BIAevaluation 4.1 software (GE Healthcare). The data were fitted into a bivalent model (2 binding sites on 1 analyte molecule bind to 2 ligand molecules) after subtraction of the RU values obtained with the reference flow cell and the buffer. A bivalent analyte gives rise to 2 sets of rate constants following the reaction equations *A* + *B* ↔ *AB* (k-forward = 2 × k_a1_; k-backward = k_d1_; KD1 = k_d1_/k_a1_) and *AB* + *B* ↔ *AB*_2_ (k-forward = k_a2_; k-backward = 2 × k_d2_; KD2 = k_d2_/k_a2_), where *A* is an analyte and *B* is a ligand. Parameters, including k_a_ (k_a1_ and k_a2_), k_d_ (k_d1_ and k_d2_), *R*_max_, and *tc*, were fitted globally, whereas *RI* was fitted locally, and concentration was fitted as a constant with bivalent binding model. Mass transfer and the closeness of the fit were assessed by *tc* and χ^2^ values, respectively.

Competition binding assays were performed out in triplicate with varying concentrations (1–100 mM) of lactose (negative control), 6’-SL (Carbosynth), and SA (Carbosynth). Briefly, 50–200 nM gp120/gp145/Soc–V1V2 or 50 nM Siglec-1 were injected in the presence or absence of lactose, 6′-SL, or SA onto immobilized Siglec-1 or gp120/gp145/Soc-V2 proteins using similar buffer conditions as mentioned above. The data analysis was performed with BIAevaluation 4.1 software, as mentioned above.

### RNA extraction and qRT-PCR for viral entry

RNA was extracted from GM-CSF–derived or M-CSF–derived MDMs infected with HIV-1 (BaL, subtype B) using the RNeasy Mini Kit and QIAshredder (Qiagen). The RNA was eluted in RNase free water, and the concentration was determined with a Nanodrop 2000 (Thermo Scientific). The qRT-PCR reactions were performed using the TaqMan RNA-to-Ct Master Mix and the Viia7. Reactions (50 μl) were performed in the presence of the master mix, 0.2 μM each of *gag* forward and reverse primers (5′-CATGTTTTCAGCATTATCAGAAGGA-3′; 5′-TGCTTGATGTCCCCCCACT-3′), *gag* probe (5′-6FAM- CCACCCCACAAGATTTAAACACCATGCTAA-TAMRA-3′), and 1X human GAPDH VIC-TAMRA. Cycling parameters were 48°C for 20 min, 95°C for 10 min, then, 45 cycles at 95°C for 15 s, and 59°C for 1 min. ΔCt values were calculated to normalize the HIV gag level as a function of the GAPDH/cellular RNA signal. When comparing the GM-CSF–derived vs. the M-CSF–derived MDMs for viral entry, the ΔΔCt values were determined by ΔΔCt = (ΔCt of GM-CSF sample) – (ΔCt of M-CSF sample). The percentage of inhibition at entry was based on comparative qPCR analysis in the presence of the inhibitor vs. in the absence of the inhibitor (PBS). When examining the possible inhibitors of entry, the ΔΔCt values were determined by ΔΔCt = (ΔCt of PBS sample) – (ΔCt of inhibitor sample). Fold-difference was calculated with 2^−∆∆Ct^. The percentage of inhibition was calculated based on the equation −([Fold-difference] − 1) × 100.

### DNA extraction and qPCR

DNA from 0.15–1.5 × 10^6^ GM-CSF–derived or M-CSF–derived MDMs infected with HIV-1 (BaL or US-1, both subtype B) or with medium was extracted using cell lysis buffer (10 mM Tris-HCl [pH8], 1 mM EDTA, 0.001% Triton X-100, 0.001% SDS, and freshly added 1 μg/ml proteinase K) at 30 μl per 1 × 10^6^ cells, followed by successive incubations at 60°C and 95°C for 60 min and 15 min, respectively. Relative multiplex DNA target levels were determined using 2X TaqMan Universal PCR Master Mix and the 7500 Real Time PCR System (Applied Biosystems). Briefly, samples from both a short BaL virus infection time course (0.25–0.5 h) composed of 3 donors in triplicate and a long US-1 virus infection time course (1–96 h) composed of 2 donors in duplicate were processed in 30 μl volumes inclusive of 1 μl of neat lysate. Two primer/probe (dual 5′ FAM- or VIC- and 3′ TAMRA-labeled) sets, differing only in the 3′ primer, were used to differentiate between HIV entry and replication: 1) HIV strong stop (measure of early reverse transcripts, virus entry): 5′R (US-1) (5′-AACTAGGGAACCCACTGCTTAA), 3′U5 (5′-TGAGGGATCTCTAGTTACCAGAGTCA), and R-probe (5′-FAM-CCTCAATAAAGCTTGCCTTGAGTGCTTCAA-TAMRA); and 2) HIV long terminal repeat–*gag* (measure of late reverse transcripts, replication): 5′R (US-1), 3′*gag* (5′-CGAGTCCTGCGTCGAGAGA), and R-probe. Amplifications were multiplexed with an Applied Biosystems–designed GAPDH primer/probe set to both normalize sample inputs and to serve as a template integrity control: GAPDH F (5′-ACCGGGAAGGAAATGAATGG), GAPDH R (5′-GCAGGAGCGCAGGGTTAGT), and GAPDH probe (5′-VIC-ACCGGCAGGCTTTCCTAACGGCT-TAMRA). Final HIV and GAPDH primer/probe concentrations were 100/200 and 75/100 nM, respectively. Cycling parameters were 95°C for 10 min, then 50 cycles at 95°C for 15 s, and 60°C for 1 min. Normalized relative HIV target levels were calculated as log_2_-transformed fold-differences from respective corresponding M-CSF 0.25 or 0.5 h samples values by 2^(−∆∆Ct)^, where ∆∆Ct = (∆Ct of the sample) − (∆Ct of M-CSF 0.25 or 0.5 h) and ∆Ct = (average Ct of HIV target) – (average Ct of corresponding to GAPDH).

### SGA

HIV-1–infected MDMs, cultured in M-CSF or GM-CSF media, were harvested at 16 h postinfection and then washed in TE buffer without Ca^2+^ or Mg^2+^. Cell lysis buffer (50 μl of 10 mM Tris-HCl containing 0.5% Nonidet P-40, 0.5% Tween 20, and 0.3 μg/ml of proteinase K was added to the cell pellet, and the mixture incubated at 55°C for 1 h to digest the cells and release DNA, and subsequently, at 85°C for 15 min to inactivate the proteinase K. An envelope gp120 nested-PCR strategy was performed on the cell lysates at dilutions that yielded 20–30% positive PCR products according to the SGA strategy [23]. The gp120 V1-V4 region nucleotide sequence corresponding to HXB2 number 6354–7485 was retrieved using SGA and sequencing. The number of sequences obtained from the virus stock, purified viruses, and GM-CSF and M-CSF cultured MDMs were 22, 21, 24, and 24, respectively.

Phylogenetic analysis was performed with MEGA 5.05 software to construct a maximum-likelihood tree with the bootstrap method at 100 replications and to calculate the mean pairwise distances. Visual inspections of the distances were also performed for evidence of the signature nucleotides between sequences from different sources. Compartmentalization of the sequences from 4 different sources was evaluated by F_ST_, SM, and AI tests using the software package HyPhy (version 2.10). *P* < 0.05 (for F_ST_ and SM) and bootstrap values >0.95 were considered statistically significant evidence of compartmentalization. For AI, 0 indicates maximum phylogenetic structure, and 1 indicates panmixia.

### DNA PCR and sequencing

The first round of PCR reagents was prepared at a final volume of 20 μl per reaction containing 10 μl of 2X GeneAMP Fast PCR Master Mix, 0.5 μl (20 mM) of the first-round primers (gp120, 5′-AGCAGAAGACAGTGGCAATGA; gp120, -3′-AGTGCTTCCTGCTGCTCC), 4 μl of water, and 5 μl of cell lysate. Forty PCR cycles (94°C for 15 s, 58°C for 30 s, and 72°C for 2 min) were performed after denaturing the DNA at 94°C for 1 min. The first-round PCR product (1 μl) was added to 19 μl of the second-round master mix, which included 10 μl of 2X GeneAMP Fast PCR Master Mix, 0.5 μl (20 mM) of the second-round primers (Z1F-TGGGTCACAGTCTATTATGGGGTACCT and JL107-GCTTTTCCTACTTCCTGCCAC) and 8 μl of water. The reaction was performed using the same cycling conditions as described for the first-round amplification. The PCR product was visualized on a 1% agarose gel containing ethidium bromide.

### Statistical analysis

Statistical analysis for cell-surface molecules and HIV-1 infection of MDM was carried out using the Mann-Whitney test (GraphPad Prism 5, version 5.0c; GraphPad Software, La Jolla, CA, USA). A 2-tailed paired *t* test was used for strong stop (HIV entry) and LTR-*gag* (HIV replication) experiments. The unpaired *t* test was used for gag RNA (HIV entry) experiments. *P* ≤ 0.05 was considered statistically significant.

## RESULTS

### M-CSF–derived MDMs show greater permissivity to HIV-1 infection

**Figure 1.**
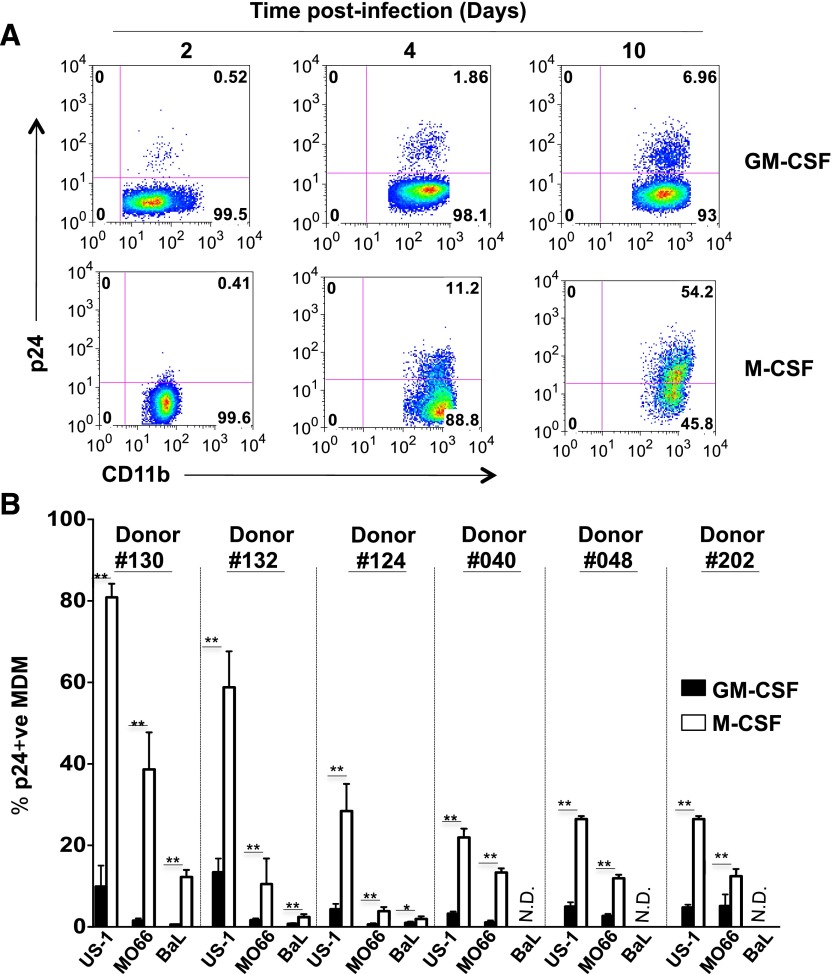
GM-CSF–derived MDM are less permissive to HIV-1 infection than M-CSF–derived MDMs. (A). Primary human monocytes were differentiated into MDMs following in vitro culture in GM-CSF or M-CSF media for 5 d and infected with US-1 (5 ng p24). Cells were harvested and analyzed for the presence of intracellular p24 by flow cytometry. Panels show dot plots of US-1–infected GM-CSF– or M-CSF–derived MDMs at 2, 4, and 10 d postinfection. A representative of 3 independent experiments is shown. (B). HIV-1 infectivity of GM-CSF– and M-CSF–derived MDMs was evaluated in 6 seronegative donors following infection with 3 primary HIV-1 subtypes (5 ng p24). Cells were analyzed on day 4 postinfection for the presence of intracellular p24 by flow cytometry. Bar graph shows the percentage (means ± sd) of infected GM-CSF– and M-CSF–derived MDMs (filled bars and open bars, respectively). Data are representative of 3 independent experiments done in triplicate. Sample sets were compared using the Mann-Whitney test (*P = 0.01; **P = 0.002).

To evaluate the effects of GM-CSF and M-CSF on HIV-1 replication, enriched monocytes from a healthy HIV-seronegative donor were differentiated into MDMs for 5 d in media supplemented with GM-CSF (GM-CSF–derived MDM) or M-CSF (M-CSF–derived MDM) and infected with HIV-1 (US-1, R5 tropic, subtype B). The percentages of infected MDMs were compared by flow cytometry on days 2, 4, and 10 postinfection (**Fig. 1A**). Because CD14 was down-regulated on GM-CSF–derived MDMs, infected MDMs were expressed as p24^+^ cells within the gated CD11b population. At day 2 postinfection, there were no significant differences in the percentages of infected MDMs in either the GM-CSF– or the M-CSF–derived MDMs. As infection progressed (day 4 and day 10), intracellular p24 in M-CSF–derived cells was significantly greater than in the GM-CSF–derived MDMs (Fig. 1A). The mean percentage of infected cells at day 4 postinfection was 2.26% in the GM-CSF–derived MDM compared with 12.52% in the M-CSF–derived MDMs (*P* < 0.002). This difference was even greater on day 10 postinfection (6.70% vs. 57.09%, *P* < 0.002), respectively. This reflected significantly higher HIV-1 replication in M-CSF compared with GM-CSF–derived MDMs between days 2 and 10 postinfection, respectively (102-fold vs. 12-fold). To further determine whether this observation was virus and/or donor restricted, GM-CSF– and M-CSF–derived MDMs from 6 different donors were infected with 2 additional HIV-1 primary viruses—BaL (subtype B) and MO66 (subtype A/E)—and analyzed on day 4 postinfection (Fig. 1B). Although different degrees of infectivity were observed with the 6 donors, a significantly higher percentage of infected MDMs was observed in the M-CSF–derived MDMs from all 6 donors and with all 3 viruses. Therefore, these data indicated that HIV-1 replication was increased in M-CSF– vs. GM-CSF–derived MDMs and was independent of virus or donor.

### Similar virus variants are present in GM-CSF– and M-CSF–derived MDMs

To determine whether the observed difference in permissiveness to HIV-1 infection and replication was due to a selection of different virus variants in the infected cells, US-1 (R5, subtype B) infected GM-CSF– and M-CSF–derived MDMs were sequenced at 16 h postinfection. The gp120, V1–V4 region, nucleotide sequence, corresponding to HXB2 numbers 6354–7485, was retrieved using SGA and sequencing. The number of sequences obtained from the virus stock, purified viruses, and GM-CSF– and M-CSF–cultured MDMs were 22, 21, 24, and 24, respectively. Phylogenetic analysis showed that the sequences from virus stock, purified viruses, and viruses from M-CSF– and GM-CSF–derived MDMs, respectively, had mean pairwise distances (means ± sd percentages) of 0.29 ± 0.16%, 0.26 ± 0.19%, 0.23 ± 0.16%, and 0.29 ± 0.15% (Supplemental Fig. 1). The *P* values for SM tests ranged from 0.445 to 0.878 with the migration events from 15–18. The F_ST_ results ranged from 0.069 to 0.816, indicating no significant differences between the sequences from the 4 sources. The AI values ranged from 0.772 to 0.995. The bootstrap values ranged from 0.36 to 0.69, which further confirmed that there was no evidence of compartmentalization among the sequences. Furthermore, no signature nucleotide or nucleotides or significant differences were identified between the virus stock and viruses obtained from GM-CSF– and M-CSF–cultured MDMs. In conclusion, there were no significant differences between the GM-CSF and M-CSF virus sequences and the virus stock. The difference in permissiveness to HIV-1 replication of the GM-CSF– compared with the M-CSF–cultured cells was, therefore, not caused by the presence of selective virus variants but was due to the cell-culture conditions.

### GM-CSF–derived MDMs are more resistant to HIV-1 entry and replication

**Figure 2.**
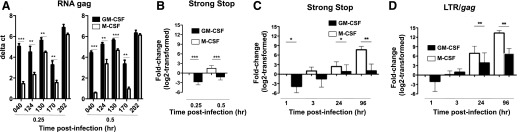
HIV-1 entry and replication is significantly decreased in GM-CSF–derived, compared with M-CSF–derived, MDMs. GM-CSF– or M-CSF–derived MDMs (filled or open bars), were infected with BaL (A and B) or US-1 (C and D). (A) RNA from the lysates at 0.25 and 0.5 h postinfection was subjected to qRT-PCR for HIV-1 gag RNA (virus entry). ΔCt values for HIV gag RNA was normalized to GAPDH RNA. A lower ΔCt value indicates a higher amount of viral RNA or higher viral entry. Lysates collected at 0.25, 0.5, 1, 3, 24, and 96 h postinfection were analyzed for early strong stop or late LTR-gag DNA reverse transcripts indicative of virus entry (B, C) or replication (D). GAPDH-normalized target levels are displayed relative to the corresponding 0.25 h sample for (B) or to 1 h samples (C and D) of M-CSF differentiated MDM as mean log2-transformed fold-change ± S.D. for either 3 or 2 iterations of 3 or 2 independent experiments (B). (*P = 0.01; **P = 0.001; ***P = 0.0001; 2-tailed paired *t* tests).

The lower intracellular p24 levels present in infected GM-CSF–derived MDMs may largely reflect a reduced efficiency in HIV-1 entry and/or subsequent replication. To differentiate between these possibilities, lysates from HIV-1–infected (BaL or US-1, subtype B) GM-CSF– and M-CSF–derived MDMs harvested over varying time periods were subjected to qPCR analysis, which is more sensitive than detection of intracellular p24 levels by flow cytometry. HIV-1 entry was evaluated with 2 different assays. qRT-PCR was used to detect the presence of gag RNA sequences, whereas the presence of R-U5 LTR cDNA sequences (HIV-1 strong stop targets) and LTR-*gag* (HIV-1 replication) sequences were coevaluated using relative qPCR. The RNA and DNA inputs were normalized based on the GAPDH housekeeping gene for each donor. To determine differences in virus entry early during infection, GM-CSF– and M-CSF–derived MDMs were infected with HIV-1 for 0.25 or 0.5 h. Lysates were harvested, and RNA was isolated and subjected to qRT-PCR (**Fig. 2A**), or the lysates were directly processed for qPCR (Fig. 2B). The data are plotted as ΔCt values in Fig. 2A. The lower the ΔCt value was, the higher was the amount of viral RNA inside the cells. As shown in Fig. 2A, HIV-1 entry was lower in the GM-CSF–derived (higher Ct value), compared with the M-CSF–derived MDMs (lower Ct value) in all 5 donors, and this difference was significant in 4 donors (donors 40, 124, 130, and 170) at both 0.25 and 0.5 h postinfection.

Independent early time courses, harvested at 0.25 and 0.5 h time points, were assessed for HIV strong stops. HIV-1 strong-stop targets, indicative of virus entry, were detected by the presence of R-U5 LTR cDNA sequences, whereas 2-LTR targets, indicative of virus replication, were detected by the presence of LTR-*gag* DNA sequences. GAPDH-normalized target levels are displayed, relative to those of the M-CSF differentiated MDMs, at the corresponding 0.25 h sample as log_2_-transformed fold-change means ± SD for 3 iterations of 3 experiments (Fig. 2B). Sample sets were compared using 2-tailed paired *t* tests (***P = 0.0001). Both 0.25 and 0.5 h time points showed increased strong stop targets over time in both MDM populations, with significantly less target detection in the GM-CSF–derived MDMs. Fold-differences in detected strong stop targets between GM-CSF– and M-CSF–derived MDMs were 7.1 and 6.9, respectively (Fig. 2B). The 2-LTR HIV-1 DNA targets were not consistently detected, indicating that either no HIV-1 replication occurred that early or that the levels were below detection. The uninfected HIV^−^ controls produced no strong stop amplification signals.

In separate experiments, lysates were collected at 1, 3, 24, and 96 h postinfection and subjected to relative qPCR analysis to detect early strong-stop reverse transcripts (Fig. 2C) and late LTR-gag DNA reverse transcripts (Fig. 2D). Mock infection samples were negative through the 96-h time point (data not shown). GAPDH-normalized target levels are displayed relative to those of the M-CSF–differentiated MDMs at the corresponding 1 h samples as log_2_-transformed fold-change means ± SD for 2 iterations from each set of 2 independent experiments (Fig. 2C and D). Sample sets were compared using 2-tailed paired *t* tests (*P = 0.01, **P = 0.001). HIV-1 strong-stop targets increased steadily in both MDM populations, but were significantly lower in the GM-CSF–derived MDMs at all time-points (Fig. 2C). Differences in detected strong-stop DNA between the GM-CSF– and M-CSF–derived MDMs at 1, 3, 24, and 96 h time points were 15.1-, 5.6-, 2.7-, and 99.6-fold, respectively (Fig. 2C). Correspondingly, the presence of 2-LTR HIV-1 DNA, which is indicative of virus replication, increased steadily in both MDM populations. However, at the later time points (24 h and 96 h) postinfection, it was significantly less in the GM-CSF–, compared with the M-CSF–derived, MDMs (*P* < 0.001). Differences for 2-LTR HIV-1 levels between the GM-CSF– and M-CSF–derived MDMs at 1, 3, 24, and 96 h were 4.2-, 0.6-, 8.2-, and 93.5-fold, respectively (Fig. 2D). The uninfected HIV^−^ controls produced no HIV-1 RNA gag signals. Collectively, these data suggest that the significantly decreased level of HIV-1 replication in the GM-CSF–derived MDMs may be the result of reduced HIV-1 entry. In each case, sample sets were compared using 2-tailed paired *t* tests (*P = 0.01, **P = 0.001, ***P = 0.0001).

### GM-CSF–derived MDMs express fewer Siglec-1 receptors

**Figure 3.**
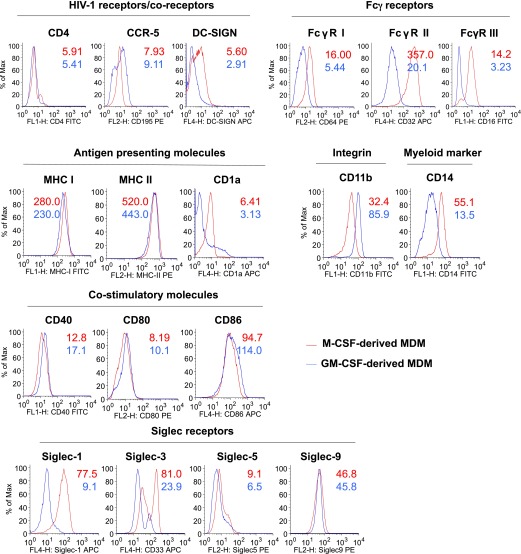
Expression of cell surface molecules on GM-CSF– and M-CSF–derived MDMs. Primary human monocytes from an HIV-seronegative donor were differentiated into MDMs following in vitro culture in GM-CSF (blue line) or M-CSF (red line) media for 5 d. Cells were harvested, stained, and the expression of cell surface molecules was analyzed by flow cytometry. The numbers in the histograms represent the MFIs of the indicated receptors. A representative histogram of 3 independent experiments performed in triplicate is shown. DC-SIGN, dendritic cell–specific intercellular adhesion molecule 3-grabbing nonintegrin; max, maximum.

The role of CD4 [24] and chemokine receptors [25] as cell-surface receptors for HIV-1 entry into target cells has been extensively documented. The differences in HIV-1 replication between GM-CSF–derived MDMs and their M-CSF–derived counterparts may be largely due to differential expression of cell-surface receptors used by HIV-1 to enter into target cells. Therefore, we evaluated the cell-surface expression of HIV-1 receptors, Fcγ receptors, antigen-presenting molecules, integrin receptors and myeloid markers, costimulatory molecules, and Siglec receptors on uninfected MDMs from 4 donors on day 5 after in vitro differentiation with GM-CSF and M-CSF. Representative histogram plots of 1 donor are shown in **Fig. 3**, and the average of the MFIs of the 4 donor cells is shown in **Table 1**. Minor differences were noted in the MFIs of CD4, CCR5, antigen-presenting molecules, costimulatory molecules, and Siglec-9 receptors on M-CSF–derived MDMs compared with GM-CSF–derived MDMs. Although CD4 expression was detected in both MDM populations, this was not due to contamination of T cells, as shown by the absence of CD3 staining (data not shown). Slight differences in the surface expression of DC-SIGN, CD64, CD11b, Siglec-3, Siglec-5, and CD32 were observed in the M-CSF–. compared with GM-CSF–derived MDMs. In contrast, there was a 5.5-fold increase in the expression of Siglec-1 (CD169) receptors in the presence of M-CSF compared with GM-CSF. Recently, the importance of Siglec-1 in HIV-1 capture and transmission by DCs [13, 17] and their ability to facilitate HIV-1 infection of macrophages [15] has been described. Therefore, we quantified the number of Siglec-1 receptors/cell on GM-CSF– and M-CSF–derived MDMs in 3 donors. In addition, we also performed similar analyses for CCR-5 and CD4 receptors. As shown in **Table 2**, the mean number of Siglec-1, CCR-5, and CD4 receptors was lower in the GM-CSF– compared with the M-CSF–derived MDMs.

**TABLE 1.** MFIs of cell-surface molecules

Surface molecule	M-CSF–derived MDMs	GM-CSF–derived MDMs
CD4	7.20 ± 0.67	6.15 ± 0.26
CCR5	8.51 ± 0.59	10.11 ± 0.65
DC-SIGN	7.12 ± 0.49	3.05 ± 0.69
CD16	13.90 ± 1.38	3.61 ± 0.51
CD32	258.00 ± 65.15	178.10 ± 73.48
CD64	15.81 ± 0.72	6.45 ± 0.72
CD40	12.72 ± 1.39	18.11 + 1.70
CD80	10.15 ± 0.74	11.80 ± 0.62
CD86	98.51 ± 1.16	112.93 ± 5.08
MHC I	301.10 ± 13.11	265.51 ± 11.12
MHC II	490.82 ± 16.93	465.54 ± 12.35
CD1a	10.25 ± 1.91	8.83 ± 0.82
CD11b	44.95 ± 5.42	83.53 ± 4.47
CD14	79.15 ± 12.11	16.48 ± 2.13
Siglec-1	88.83 ± 9.57	16.28 ± 4.64
Siglec-3	86.00 ± 6.14	23.10 ± 4.24
Siglec-5	15.83 ± 6.51	8.80 ± 1.31
Siglec-9	51.83 ± 7.20	47.05 ± 4.68

The MFI of cell-surface markers was determined by flow cytometry. Values are from triplicate samples (means ± sd) from M-CSF– and GM-CSF–derived MDMs from 4 different donors.

**TABLE 2.** Number of cell-surface molecules

Surface molecule	Donor No.	M-CSF–derived MDM	GM-CSF–derived MDM
**Siglec-1**	124	67,879 ± 5,856	10,872 ± 3,286
	130	79,190 ± 1,011	33,084 ± 824
	202	32,966 ± 6,536	14,479 ± 469
**CCR-5**	124	70,601 ± 5,784	14,902 ± 614
	130	29,690 ± 350	14,021 ± 277
	202	29,861 ± 1,067	25,542 ± 419
**CD4**	124	92,719 ± 3,523	38,034 ± 1,158
	130	68,064 ± 3,220	50,286 ± 1,639
	202	36,458 ± 1,322	30,808 ± 1,556

The number of cell-surface molecules was determined by flow cytometry. Values are from triplicate samples (means ± sd) from M-CSF– and GM-CSF–derived MDMs from 3 different donors.

### Siglec-1 binds HIV-1 and facilitates entry and infectivity of GM-CSF– and M-CSF–derived MDMs

**Figure 4.**
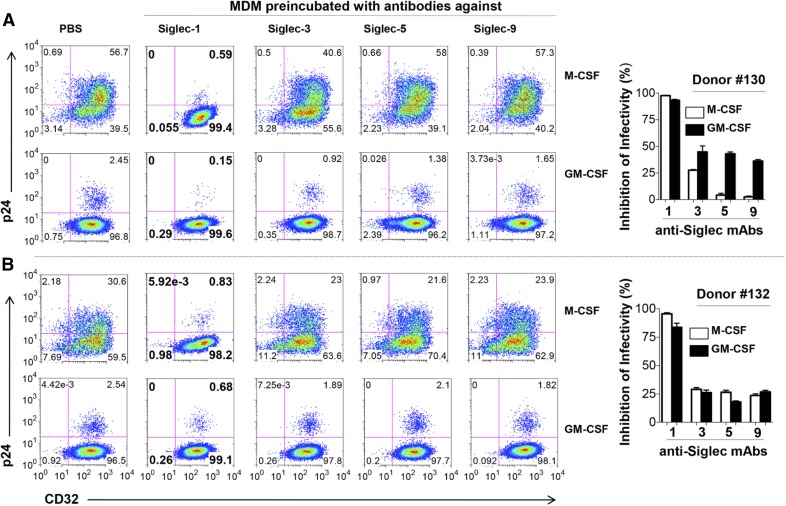
Blocking Siglec-1 receptor significantly decreases HIV-1 infectivity. Primary human monocytes were differentiated into MDM following in vitro culture in GM-CSF or M-CSF media. Triplicate wells of MDMs from donor 130 (A) and donor 132 (B) were preincubated with purified mAbs specific for the Siglec receptors (Siglec-1, -3, -5, or -9) and subsequently infected with US-1 (5 ng p24). MDMs preincubated with PBS served as infection controls. Cells were harvested on day 4 postinfection and analyzed for the presence of intracellular p24 by flow cytometry. Panels show dot plots of US-1–infected GM-CSF– or M-CSF–derived MDMs. Values in the upper right quadrants represent the percentage of infected MDMs. The data from Figs. 4A and B are represented as bar graphs (right side), and shows the percentage inhibition of infectivity (means ± sd) in GM-CSF–derived MDMs (filled bars) or in M-CSF–derived MDMs (open bars). Data are representative of triplicate wells from 3 independent experiments.

To evaluate the involvement of the various Siglec receptors in HIV-1 infection, GM-CSF– and M-CSF–derived MDMs from 2 donors (donors 130 and 132) were preincubated with purified mAbs to block Siglec-1, -3, -5, or -9 and then infected with HIV-1 (US-1, subtype B). Consistently, in both donors (donors 130, **Fig. 4A**; and 132, Fig. 4B; dot plots and their respective bar graphs), blocking Siglec-1 significantly decreased HIV-1 infection in both GM-CSF– (*P* = 0.008) and M-CSF–derived MDMs (*P* = 0.007) by 80–97%. Blocking the other Siglec molecules (Siglec-3, -5, or -9) resulted in 5–28% and 12–48% reduction of HIV-1 infection for M-CSF– and GM-CSF–derived MDMs, respectively (Fig. 4A and B). These results indicate that, compared with the other Siglec molecules, Siglec-1 was the predominant receptor involved in MDM/HIV interactions.

**Figure 5.**
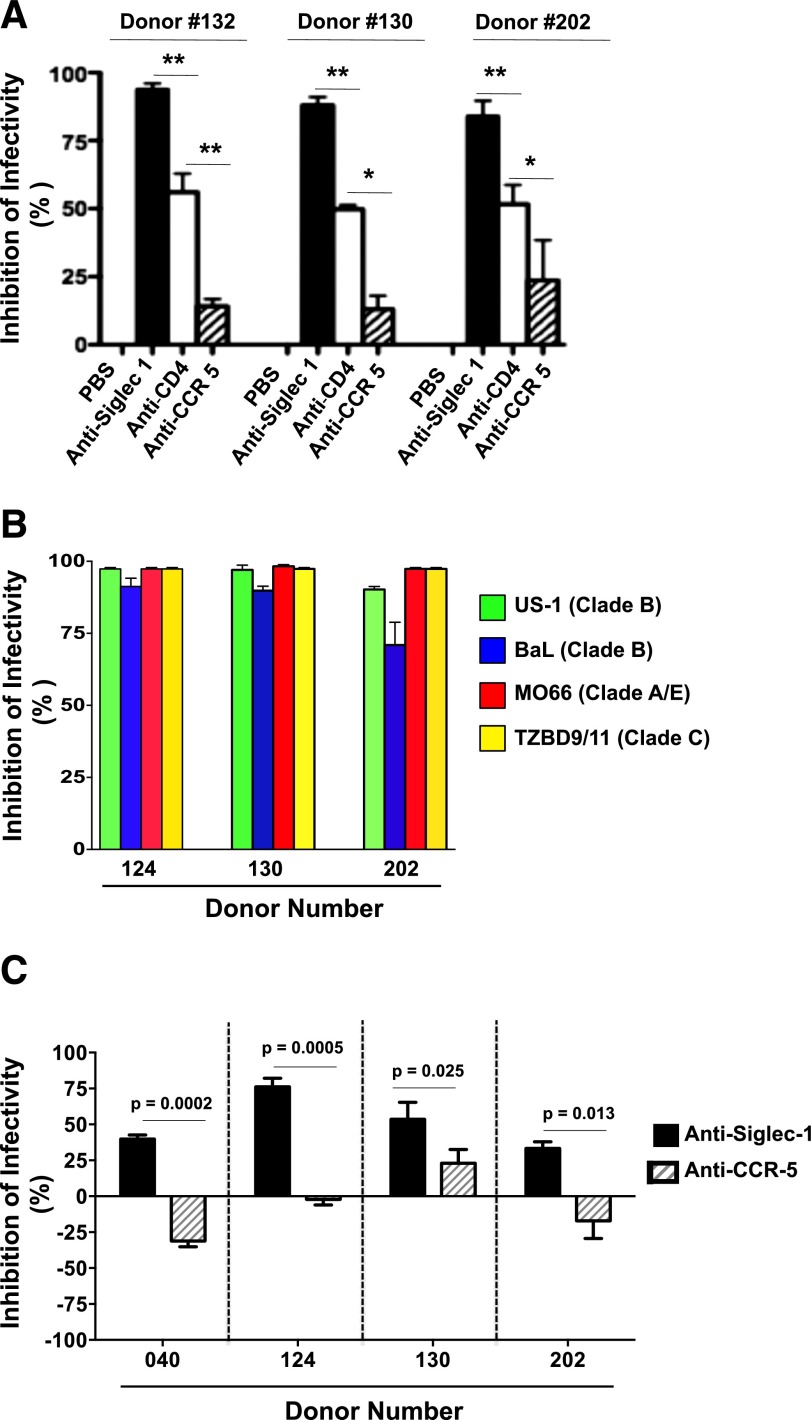
Siglec-1 is a major HIV-1 receptor on MDMs. Triplicate wells of M-CSF–derived MDMs were preincubated with PBS or with mAbs specific for Siglec-1, CD4, or CCR5 and were subsequently infected for 90 min with either BaL (A), with different virus clades (B), or for 30 min with US-1 (C). (A) Bar graphs show the percentage inhibition of infectivity (means ± sd) with anti-Siglec-1 mAbs (filled bars) or with anti-CCR5 mAbs (hatched bars). (B). Bar graph shows percentage inhibition of infectivity (means ± sd) with 4 primary viruses belonging to subtypes B (US-1, BaL), A/E (MO66), and C (TZBD9/11) with anti-Siglec-1 mAbs. (C) Bar graph shows percentage inhibition of virus entry (means ± sd) with anti-Siglec-1 mAbs (filled bars) or with anti-CCR5 mAbs (hatched bars). Data are representative of 3 independent experiments. The percentage inhibition of virus entry was calculated as described in the “Materials and Methods.”

To further compare the involvement of Siglec-1 and other well-characterized HIV-1 receptors (CD4 and CCR5 molecules) involved in HIV-1 infection, M-CSF–derived MDMs from 3 donors (donors 132, 130, and 202) were evaluated for HIV-1 infectivity (**Fig. 5A**). In all 3 donors, blocking Siglec-1 receptor decreased HIV-1 infection by 84–94% (*P* = 0007). In all 3 donors, blocking the CD4 receptor resulted in a 50–56% decrease in HIV-1 infectivity. In contrast, blocking the CCR5 receptor resulted in only a minimal reduction in HIV-1 infectivity in all 3 donors (Fig. 5A).

The effect of anti-Siglec-1 mAb was further evaluated in 3 donors with 4 viruses: US-1 (subtype B), BaL (subtype B), MO66 (subtype A/E), and TZBD9/11 (subtype C) (Fig. 5B). Anti-Siglec-1 inhibited the infectivity of all 4 viruses tested in all 3 donors, with inhibition ranging from 71–98%. To determine whether anti-Siglec-1 and anti-CCR5 mAbs would block HIV-1 entry, M-CSF–derived MDMs from 4 donors were preincubated with the mAbs and subsequently infected with HIV-1 BaL (subtype B) for 30 min. RNA isolated from the lysates was analyzed by qRT-PCR for the presence of gag RNA sequences. In contrast to anti-CCR5 mAbs, anti-Siglec-1 mAbs significantly inhibited virus entry into the MDMs of all 4 donors (Fig. 5C).

These data demonstrate the involvement of Siglec-1 during HIV-1/MDM interaction and further show that its effects are independent of both donor and virus. It is plausible that the Siglec-1/HIV interaction is a significant process during the initial cell/virus encounter, and the efficiency of this interaction may subsequently influence the outcome of HIV entry and replication in MDMs.

### Siglec-1 binds to the V1V2 region of the HIV envelope

**Figure 6.**
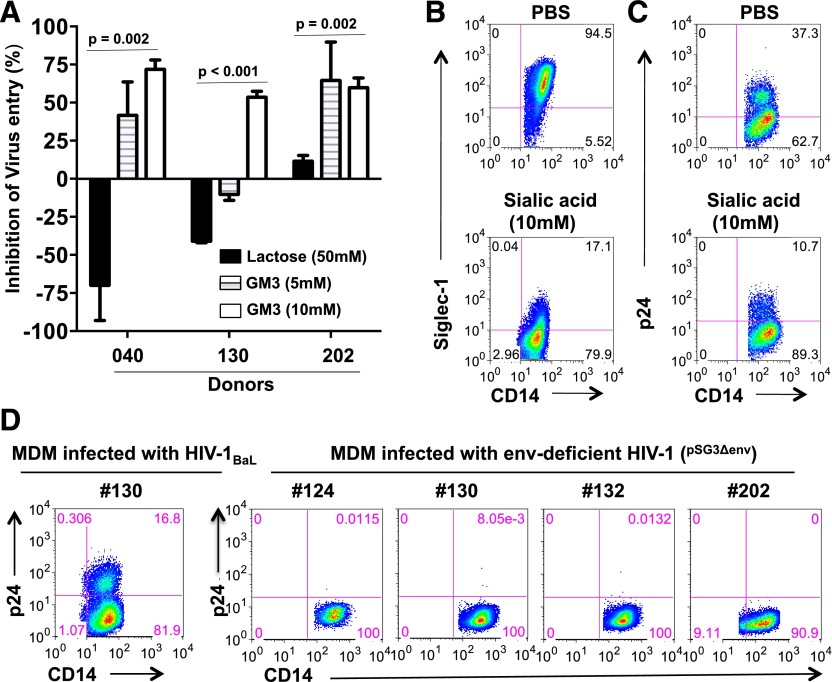
Blocking Siglec-1 receptor with sialyllactose or sialic acid decreases entry and replication of HIV-1. Triplicate wells of M-CSF–derived MDMs were preincubated with PBS (A), lactose, or 2′,3′siallylactose (GM3), or PBS or SA (B and C) for 30 min at room temperature. (A) Cells were washed and infected with US-1 for 30 min, lysed, and the lysates were analyzed for the presence of HIV-1 gag RNA by qRT-PCR. Data are representative of triplicate wells. Bar graph show percentage inhibition of virus entry (means ± sd. (B) MDMs were stained and analyzed for expression of Siglec-1. A representative of 3 independent experiments is shown. (C) The cells were infected with US-1 for 90 min and analyzed on day 4 for the presence of intracellular p24. Data are representative of 2 independent experiments. (D) MDMs were infected with BaL or with envelope (env)-deficient HIV-1 (^pSG3Δenv^) for 90 min and analyzed on day 4 for the presence of intracellular p24. Panels show dot plots of MDMs infected with BaL or with env-deficient HIV-1 (^pSG3Δenv^).

Glycans on HIV-1 gp120 bind Siglec receptors to gain entry into MDMs [15]. Therefore, we hypothesized that SA would block Siglec-1 and subsequently modulate HIV-1 infection in M-CSF–derived MDMs. M-CSF–derived MDMs from 3 donors were preincubated with 2′,3′sialyllactose (GM3) or with lactose and then infected with BaL. In contrast to lactose, in all 3 donors, HIV-1 entry was significantly inhibited by GM3 (**Fig. 6A**). Using flow cytometry, we observed that incubation with 10 mM SA reduced the number of Siglec-1 receptors detected on the cells from 95 to 17% (Fig. 6B), probably from masking the receptor, which in turn led to a corresponding decrease in HIV-1 infection (Fig. 6C). To show that virus infectivity was mediated by its envelope, M-CSF–derived MDMs were infected with HIV-1 (BaL, subtype B) or as a control with envelope-deficient HIV-1 (pSG3^Δenv^). Cells were harvested on day 4 postinfection and analyzed for the presence of intracellular p24 by flow cytometry (Fig. 6D). As expected, the envelope-containing virus showed that approximately 17% of the cells were infected. In contrast, none of the 4 donors infected with the envelope-deficient virus showed any infectivity, thus confirming the requirement of the envelope protein for infectivity.

**Figure 7.**
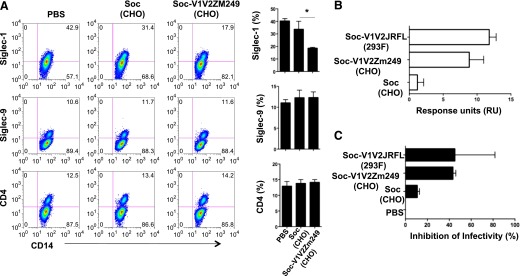
V1V2 protein binds specifically to Siglec-1. (A) Triplicate wells of M-CSF–derived MDMs were preincubated with recombinant CHO-expressed Soc–V1V2Zm249 (CHO) envelope protein, Soc (CHO), or PBS for 30 min at room temperature, and the expression of Siglec-1, Siglec-9, and CD4 was analyzed by flow cytometry. Bar graphs show percentage of expression (means ± sd) of the cell receptors. A representative of 2 independent experiments is shown (*P = 0.02, Mann Whitney test). (B) Siglec-1 was immobilized on a CM5 biosensor chip and the binding (RUs) of CHO-expressed Soc (CHO), Soc-V1V2Zm249 (CHO), or 293F-expressed Soc-V1V2JRFL (293F) recombinant Soc–V1V2 proteins was determined by SPR. (C) MDMs were preincubated with PBS or with recombinant Soc-V1V2 proteins for 30 min at room temperature and infected with HIV-1. Cells were harvested on day 4 postinfection and analyzed for the presence of intracellular p24 by flow cytometry. Bar graphs show the percentage inhibition of infectivity (means ± sd). Data are representative of 2 independent experiments.

Complex carbohydrates with terminal SA residues are present in the *N*-linked glycosylation sites of the 5 variable regions (V1–V5) of gp120 [26] and facilitate HIV-1 binding and infection of target cells [27, 28]. Our data demonstrate that Siglec-1 is a major receptor for HIV-1 entry on MDMs. Because the V2 loop is the most accessible region of the viral envelope at the tip of the trimer, and although there are no CD4 contact residues in V1V2 loop, it is possible that the V1V2 configuration may sterically affect the accessibility of the envelope protein to CD4 on T cells. Therefore, we examined whether scaffolded 293F or CHO-expressed V1V2 proteins would bind to Siglec-1. We hypothesized that if the recombinant V2 proteins bound to Siglec-1, Siglec-9, or CD4, those receptors would be blocked, thus resulting in decreased detection (specific staining) of those molecules. As shown in **Fig. 7A** (dot plots and bars graphs), preincubation of MDMs with Soc- (bacteriophage T4 protein) scaffolded CHO expressed V1V2 proteins from a transmitted/founder subtype C virus (Soc-V1V2Zm249 protein), which resulted in a significant decrease in Siglec-1 staining (17.9%) on the MDMs (*P* = 0.02) compared with MDMs preincubated with the Soc protein (31.4%) or PBS (42.9%). Similar results were obtained with 293F-expressed V1V2Zm249 protein scaffolded with another bacteriophage protein gp16 (data not shown). The recombinant envelope proteins had no effect on the staining of Siglec-9 or CD4 on the surface of MDMs, thereby demonstrating that the V1V2 proteins specifically bound to Siglec-1. The decreased detection of Siglec-1 after incubation with SAs or Soc-scaffolded V1V2 proteins could be due to masking of the anti-Siglec-1 mAb-binding site on the receptor.

The interaction of the V1V2 proteins with Siglec-1 was further evaluated using an SPR assay (Fig. 7B, and Supplemental Table 1). Three different V1V2 proteins (transmitted/founder subtype C Soc-V1V2Zm249, subtype B Soc-V1V2WITO, and subtype B Soc-V1V2JR-FL) showed significantly more binding to immobilized Siglec-1 compared with the Soc protein alone. The extremely low level of binding by Soc alone to Siglec-1 established the specificity of binding and the interaction through the V1V2 region (Fig. 7B and Supplemental Table 1). Although the glycosylation patterns of the CHO and 293F proteins may differ, they did not exhibit any significant differences in binding to Siglec-1. Synthetic, nonglycosylated, linear and cyclic V2 peptides did not bind to immobilized Siglec-1 (data not shown), thus signifying the importance of glycans and/or protein conformation during the binding interactions.

To determine whether binding of the recombinant V1V2 envelope proteins to Siglec-1 would modulate HIV-1 infection, MDMs were preincubated with Soc-V1V2Zm249 (CHO), Soc-V1V2JR-FL (293F), Soc (CHO-S), or PBS and were subsequently infected with US-1. As shown in Fig. 7C, preincubation of MDMs with either of the V1V2 recombinant proteins resulted in a 45% decrease in viral infection when compared with MDMs preincubated with Soc or PBS.

### Siglec-1 binds to HIV envelope proteins and to the V1V2 region with high affinity

**Figure 8.**
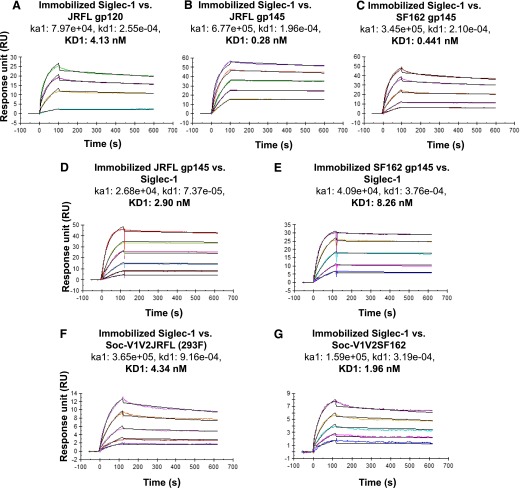
Siglec-1 binds to trimeric gp145 and to V1V2 protein with high affinity. Recombinant HIV-1 envelope proteins JR-FL monomeric gp120 (A), JR-FL FD trimeric gp145 (B), SF162 FD trimeric gp145 (C) were injected at varying concentrations (0.98–250 nM) over immobilized Siglec-1 (2500 RUs). In separate experiments, varying concentrations of Siglec-1 (7.81–250 nM) were injected over immobilized JR-FL FD trimeric gp145 (1400 RUs) (D) or SF162 FD trimeric gp145 (900 RUs) (E). Soc-V1V2JR-FL (F) or Soc-V1V2SF162 (3.91–125 nM) (G) were injected over immobilized Siglec-1 (2,500 RU). The sensorgrams show the response binding units (RU). The dissociation rate constants (K_D_) are shown. Each measurement was repeated at least 3 times.

The binding affinities of recombinant envelope proteins (293F-expressed monomeric subtype B JR-FL gp120 and trimeric JR-FL and SF162 gp145 with foldon, a trimerization domain from bacteriophage T4 fibritin), and V1V2 proteins to immobilized Siglec-1 (2500 RUs) were determined by SPR (**Fig. 8**). Kinetic studies were carried out with varying concentrations of the envelope protein. The bivalent model was the best fit for the data, indicating that there could be multiple sites on the envelope protein for binding to Siglec-1 (Fig. 8). These data showed that the recombinant envelope proteins tested were bound to Siglec-1 with low nanomolar affinity. These data showed that the binding affinities for the JR-FL and SF162 gp145 trimeric proteins to immobilized Siglec-1 (Fig. 8B and 8C) were similar and were 9- to 15-fold higher than the binding affinity to monomeric gp120 protein (Fig. 8A). In the reverse experiment, Siglec-1 did not bind to the immobilized monomeric envelope proteins. However, it bound to immobilized trimeric proteins with a 10- to 20-fold less affinity than the binding of trimeric proteins to immobilized Siglec-1 (compare Fig. 8B and C with D and E). We then determined the binding affinities of Soc-V1V2JR-FL (293F) and Soc-V1V2SF162 (CHO-S) to immobilized Siglec-1. As shown in Fig. 8F and G, Soc-V1V2JR-FL (293F) and Soc-V1V2SF162 (CHO-S) bound to Siglec-1 with much less affinity (15.5-fold and 4.5-fold, respectively) compared with the corresponding trimeric proteins. These data suggest that there might be multiple sialyl binding sites for Siglec-1 on the envelope proteins.

### Binding of monomeric and trimeric HIV-1 envelope proteins and V1V2 to Siglec-1 is inhibited by SA

**Figure 9.**
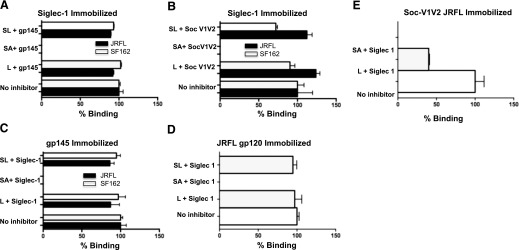
Sialic acid inhibits binding of monomeric and trimeric HIV-1 envelope proteins and V1V2 to Siglec-1. JR-FL FD trimeric gp145 and SF162 FD trimeric gp145 (50 nM) proteins (A) or Soc-V1V2JR-FL and Soc-V1V2SF162 (100 nM) (B) were incubated in the absence or presence of 6′-sialyllactose (SL, 1 mM), SA (SA, 10 mM), or lactose (L, 10 mM) and were then injected over immobilized Siglec-1 (3700 RUs). In separate experiments, Siglec-1 (50 nM) was incubated in the absence or presence of 6′-sialyllactose (SL, 10 mM), SA (SA, 10 mM), or lactose (L, 10 mM) and then injected over immobilized JR-FL FD trimeric gp145 (1400 RUs) or SF162 FD trimeric gp145 (900 RU) (C) or JR-FL gp120 (D) or Soc-V1V2JR-FL (E). The data are represented as bar graphs and show the percentage binding (means ± sd) of triplicate measurements as determined by SPR analysis.

To test the hypothesis that the interaction of Siglec-1 to gp120/gp145/Soc-V1V2 takes place through SA-binding sites, we examined 293F-produced gp120 (200 nM), trimeric gp145 (50 nM), 100 nM Soc-V1V2JR-FL (293F), or Soc-V1V2SF162 (CHO) in the absence or presence of varying concentrations of sialyllactose (1, 5, 10 and 16 mM) or SA (1, 5, 10, 50 and 100 mM) with immobilized Siglec-1 (**Fig 9A** and **B**) using SPR. In the reverse experiment, we incubated 50 nM Siglec-1 with 10 mM sialyllactose, SA, or lactose, and then injected it over immobilized JR-FL gp145 (Fig. 9C), JR-FL gp120 (Fig. 9D), or Soc-V1V2JR-FL (Fig. 9E). The response units and the percentage of inhibition in the presence of 10 mM inhibitors were determined. Lactose was used as a negative control in both experiments. In both experiments, the binding of the HIV-1 envelope proteins to Siglec-1 was inhibited only in the presence of SA (Fig. 9). No inhibition was seen with sialyllactose or lactose at any of the concentrations used. These results indicate that the interaction of HIV-1 envelope proteins with Siglec-1 occurs through the SA binding sites located on gp120 and, in particular, on the V1V2 region.

### Soc–V1V2 protein inhibits HIV-1 entry and replication

**Figure 10.**
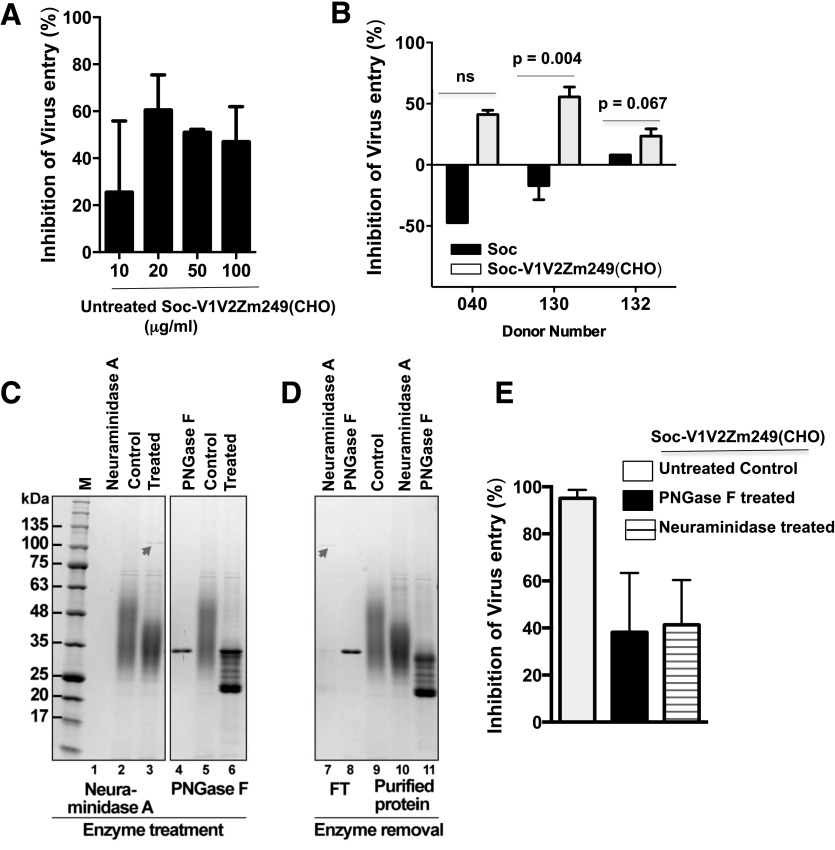
Soc–V1V2 protein inhibits HIV-1 entry. M-CSF–derived MDMs were preincubated with Soc-V1V2Zm249 (CHO) (A) or Soc-CHO or Soc-V1V2Zm249 (CHO) (B) and infected with HIV-1. Lysates were analyzed for HIV-1 *gag* RNA. (C) CHO expressed Soc-V1V2 Zm249 protein (untreated, lanes 2, 5), after treatment with (neuraminidase A, lane 3), (PNGase F, lane 6), and enzymes alone (lanes 1, 4). (D) Neuraminidase A and PNGase F are shown in the flow-through fractions (lanes 7 and 8). Bound Soc-V1V2 proteins eluted with imidazole (untreated, lane 9; neuraminidase A treated, lane 10; PNGase F treated, lane 11). Arrows show the neuraminidase A band. (E) M-CSF–derived MDMs were preincubated with untreated Soc-V1V2Zm249 (CHO) (open bars), PNGase F-treated (filled bars), or neuraminidase A-treated Soc-V1V2Zm249 (CHO) (hatched bars), infected with HIV-1 and were analyzed for the presence of intracellular p24. Bar graphs show the percentage inhibition of infectivity (means ± sd). (*P = 0.03 compared with the untreated Soc-V1V2Zm249 (CHO) protein, Mann-Whitney test).

Based on the above results, we next determined whether gp145 trimers and Soc-V1V2Zm249 protein would inhibit virus entry. As shown in Supplemental Fig. 2, JR-FL gp145 trimer inhibited the entry of BaL into M-CSF–derived MDMs in a dose-dependent manner in the 2 donors tested. Similarly, varying concentrations of Soc-V1V2Zm249 protein also inhibited the entry of BaL, with 60% inhibition observed at a concentration of 20 μg/ml of Soc-V1V2Zm249 (**Fig. 10A**). This optimum concentration was then evaluated in a subsequent experiment in 3 donors. In contrast to the Soc protein (20 μg/ml), the Soc-V1V2Zm249 protein (20 μg/ml) significantly inhibited, to varying levels, the entry of BaL into M-CSF–derived MDMs in all 3 donors (Fig. 10B).

There are at least 2–3 glycans in the V1V2 domain that contain terminal 2′,3′-SAs. As shown above, SA binds to Siglec-1 and inhibits HIV-1 infectivity (Fig. 6C). Therefore, we determined whether removal of SA from the Soc-V1V2Zm249 protein by treatment with neuraminidase A or removal of *N*-glycans with PNGase F would reduce the inhibition seen with the untreated protein (Fig. 10C, D, and E). Treatment with neuraminidase A or with PNGase F resulted in desialyation and deglycosylation as visualized by a shift in the electrophoretic mobility of the protein on SDS-PAGE with the proteins showing sharper bands (Fig. 10C, lanes 3 and 6) after enzymatic treatment compared with the diffuse bands before treatment (Fig. 10C, lanes 2 and 5). Neuraminidase A and PNGase F were removed from the reaction mixtures with Ni-NTA agarose beads, which specifically bound to His-tagged Soc–V1V2, allowing the enzymes to pass in the flow-through fractions (Fig. 10D, lanes 7 and 8). Removal of the enzymes was important because incubation of these proteins with MDMs in the presence of the enzymes could affect the cells because they have sialylated and glycosylated proteins on their cell surface. M-CSF–derived MDMs were preincubated with untreated, PNGase F–treated, and neuraminidase A–treated Soc-V1V2Zm249 protein and then infected with HIV-1. As shown in Fig. 10E, the untreated Soc–V1V2Zm249 protein inhibited viral infectivity to >95%. In contrast, this inhibition of viral infectivity was significantly reduced upon removal of SA or glycans from Soc–V1V2Zm249 by treatment with neuraminidase A or PNGase F (*P* = 0.03, in both cases) (Fig. 10E), thus showing the importance of SA residues and glycans in HIV-1 infection.

## DISCUSSION

Our study reports 2 novel findings: 1) expression of Siglec-1, an important attachment receptor for HIV-1 on myeloid cells, is differentially regulated in MDMs exposed to M-CSF or GM-CSF; and 2) SAs and the V1V2 region of the HIV-1 envelope interact with Siglec-1 on MDMs. Macrophages differentiated in the presence of M-CSF supported increased HIV-1 infection compared with GM-CSF–derived MDMs. The difference in HIV-1 infection between the 2 MDM populations was due to significantly more virus being bound to, and subsequently entering and replicating in, M-CSF–derived MDMs than in GM-CSF–derived MDMs. Importantly, there were no differences in the virus variants in the M-CSF–derived, compared to the GM-CSF–derived, MDMs with respect to the V1V2 amino acid sequence and *N*-linked glycosylation, ruling out the presence of different virus variants within the quasispecies of the virus isolates tested.

Cytokines in the environmental milieu have a critical role in the regulation of macrophage phenotypes and their responses to intracellular pathogens [29, 30]. In this study, we used GM-CSF and M-CSF to differentiate blood-enriched monocytes into macrophages. Originally defined as hematopoietic growth factors [31], evidence exists that M-CSF and GM-CSF affect host defense and inflammation [32, 33]. GM-CSF drives macrophages into M1, a proinflammatory phenotype [34] with more efficient control of intracellular bacterial [35] and HIV-1 replication [36] than M-CSF–induced M2 macrophages have [37, 38]. Our data show that HIV-1 infection is significantly reduced when macrophages were grown in a GM-CSF environment as opposed to an M-CSF environment. This observation is in agreement with other studies that reported significantly reduced HIV-1 replication in M1 macrophages, compared with M2 macrophages [36, 38]. Further analyses of the sequences of PCR products derived from HIV-1 infected macrophages that were differentiated either in M-CSF or GM-CSF revealed that similar viral sequences were indeed present in both MDM populations. This was an important observation because it ruled out the possibility of different virus variants arising from a particular macrophage phenotype as the cause of the difference in HIV replication between the macrophage populations.

We found significantly fewer viruses entered and subsequently underwent replication in the GM-CSF–derived macrophages as opposed to their M-CSF–derived counterparts, suggesting receptor–virus interaction as an important determinant for HIV-1 infection of target cells. CD4 and CCR5 are well-characterized receptors for HIV-1 entry and have been reported to function as interdependent accessory molecules for infection of target cells [39]. M-CSF has been reported to up-regulate the expression of CD4 and CCR5 receptors on macrophages [40, 41]. In contrast, GM-CSF can down-regulate expression of these receptors and drive macrophages to a proinflammatory phenotype [34]. Our data (Table 2) are in line with these published findings. Because M-CSF is the cytokine primarily produced during the steady state, it is conceivable that macrophages differentiated in vivo under these conditions may express enhanced levels of Siglec-1, CD4, and CCR5 and, therefore, display increased susceptibility to HIV-1.

Both CD4 and Siglec-1 had a profound influence on HIV-1 infectivity. Blocking Siglec-1 with specific monoclonal antibodies masked the accessibility of the receptor and resulted in the abrogation of HIV-1 infection in both GM-CSF– and M-CSF–derived macrophages. Furthermore, anti-Siglec-1 mAbs inhibited the infectivity of several primary viruses belonging to different subtypes in the different donors tested. This is in line with recent findings [13, 14, 17] that Siglec-1 is an important receptor on myeloid cells for HIV-1. Indeed, transfection of Siglec-1 into Raji B cells allowed those cells to efficiently capture HIV-1 and subsequently infect CD4^+^ T cells [17]. In vivo, Siglec-1 expression is up-regulated on monocytes during HIV-1 infection [42–44], on activated macrophages during chronic inflammation [45], and in tumors [46]. As shown in 2 recent studies [13, 17], increased surface expression of Siglec-1 on DCs following stimulation with LPS or type-1 interferon resulted in increased HIV-1 capture and *trans*-infection of T cells. Therefore, the significantly enhanced levels of HIV-1 infection in M-CSF–derived macrophages may be related to their correspondingly increased levels of Siglec-1 expression.

Our data showed a requirement for the gp120 envelope protein in virus/MDM interactions and that exposure of MDMs to GM3 inhibited viral entry. The glycans on the heavily glycosylated HIV-1 envelope protein were shown to mediate HIV-1 interaction with target cells [15, 47–49]. However, those studies did not identify the region of gp120 that interacted with Siglec-1. Studies suggest that there are at least 2–3 glycans in the V1V2 domain that contain terminal 2′,3′-SAs [50–53]. Our study shows that Siglec-1 binds to the V1V2 domain of the HIV-1 envelope protein and that this interaction is inhibited by SA.

Siglecs are transmembrane proteins that interact with sialylated ligands on cells and pathogens [16, 54]. Because the number of envelope trimers on the viral surface is limited, a high-affinity interaction between Siglec-1 and the trimer would likely be required for efficient capture, entry, and dissemination of the virus. Although there might be other regions of the envelope protein that could also bind to Siglec-1, the location of the V1V2 region exposed at the apex of the envelope trimer spike [51] is probably the most accessible for interaction with the Siglec-1 receptor [51, 53]. The unusually long, 17 extracellular Ig-like domains in Siglec-1 that extends the receptor away from the cell surface may have an important role in the pathogenesis of the infection by facilitating the efficient binding of HIV and bringing the attached virus in close proximity to the CD4 receptor for subsequent interaction and entry.

The degree of glycosylation and the types of glycans present on gp120 also affect its binding to Siglec-1. Our results show that 293F-expressed subtype B trimeric envelope protein gp145 bound to Siglec-1 with dissociation constants in the nanomolar range. Trimeric gp145 exhibited a 10- to 20-fold greater affinity than did the monomeric gp120. This is in contrast to the dissociation constants observed in the micromolar range by Zou et al. [15]. These differences could be due to different subtypes and/or due to the different experimental set up in the 2 studies. In our study, we used a lower density of the ligand. The dissociation constants for the V1V2 proteins were also in the nanomolar range but were 4.4- to 15.5-fold higher than that seen with the trimeric protein. The higher affinity of Siglec-1 to the trimeric envelope proteins indicates that the structure and conformation of the envelope protein that orients the SA residues or its ability to affect neighboring residues determines the affinity of the interaction between these 2 proteins. The bivalent model suggests the presence of multiple binding sites on the envelope protein to Siglec-1. Furthermore, trimeric gp145 and the V1V2-scaffolded protein from a transmitted/founder virus (Zm249) not only bound to Siglec-1 on MDMs but also inhibited virus entry and subsequent infectivity. Like V1V2Zm249, V1V2-scaffolded protein from a chronic virus (JR-FL) also bound to Siglec-1 and inhibited the subsequent infection of MDMs with primary HIV-1, even though the 2 proteins were produced in 2 different mammalian cell lines. Removal of SAs or glycans from V1V2Zm249 by enzymatic treatment resulted in a significant decrease in inhibition of infectivity compared with the almost 100% inhibition observed with the untreated protein.

The binding of trimeric gp145 and V1V2 proteins was specifically inhibited by SA and not by lactose. In our study, depending on the donor, HIV-1 entry was inhibited 50–75% in the presence of 2′,3′-sialyllactose (GM3), whereas 0–10% inhibition was observed in the presence of lactose. These data indicate not only the specificity of the interaction but also suggest that Siglec-1 may exist in an unmasked state. In contrast, Siglec-9, although having a high-affinity receptor for SA, exhibited 5-fold less binding to the trimeric protein and 3-fold less binding to V1V2 proteins, suggesting that the Siglec-9 receptors on the cell surface may be in a masked state. Previous studies have shown that Siglec-9 displayed significant adhesion only after neuraminidase treatment [15].

In conclusion, M-CSF increased the surface expression of Siglec-1, which rendered the macrophages more permissive to HIV-1 infection by promoting the interaction of Siglec-1 with the V1V2 region of gp120 and its associated SAs. HIV-1 can use Siglec-1 and other host receptors for attachment, and the degree of expression of these receptors may be influenced by the microenvironment. In conjunction with antiretroviral therapy, antibodies that block Siglec-1 and HIV-1 envelope interaction may provide a new way of further preventing HIV-1 infection. These aspects should be considered during HIV-1 vaccine design and development.

## AUTHORSHIP

O.J., H.V.T., and M.R. conceived and designed the experiments. O.J., H.V.T., J.K., W.A., P.K.E., and K.K.P. performed the experiments. O.J., H.V.T., P.K.E., S.T., and V.B.R. analyzed the data. O.J., H.V.T., J.H.K, N.L.M., C.R.A., V.B.R., and M.R.. discussed the data. W.A., G.G., R.T., and V.B.R contributed reagents/materials/analysis tools or provided helpful suggestions. O.J. and M.R. wrote the paper with contributions from all authors.

## DISCLAIMER

The views expressed in this article are those of the authors and do not necessarily reflect the official policy or position of the Department of the Army, Department of Defense, nor the U.S. Government.

## Abbreviations

6′-SL: 2,6′-sialyllactose
AI: Simmonds Association Index
CHO: Chinese hamster ovary
Ct: cycle threshold
DC: dendritic cell
FD: foldon
F_ST_: Wright’s measure of population subdivision
GM3: sialyllactose
LTR: long terminal repeat
M-CSF: macrophage colony-stimulating factor
MDM: monocyte–derived macrophage
MFI: mean fluorescent intensity
Ni-NTA: nickel–nitrilotriacetic acid
PNGase F: peptide *N*-linked glycosidase F
qPCR: quantitative polymerase chain reaction
qRT-PCR: quantitative reverse transcriptase-polymerase chain reaction
RU: response unit
SA: sialic acid
SGA: single-genome amplification
Siglec: Sialic acid-binding immunoglobulin-like lectin
SM: Slatkin-Maddison test
Soc: small outer capsid protein
SPR: surface plasmon resonance
WRAIR: Walter Reed Army Institute of Research
